# Morphinism among Physicians

**Published:** 1899-12

**Authors:** 


### Morphinism Among Physicians.

It is to be hoped that the figures cited by Dr. T. D. Crothers at the recent meeting of the New York Medical Association, to the effect that 10 percent of medical men are morphin habitues, are exaggerated, although there are few individuals so much exposed to the danger of addiction as they, by reason of the nature of their work, with its cares, responsibilities and emotions, the necessary and often unavoidable irregularity of their hours of sleep, eating and recreation. With the knowledge of the danger, it should be the invariable rule of physicians never to take any drug, and especially of the sedative or hypnotic class, on their own initiative, but always and only on the advice and under the observation of a trustworthy colleague. Here, perhaps, more than in any other relation, the apt truism of Weir Mitchell may be quoted as appropriate: "The physician who treats himself has a fool for a patient."—*The Journal.*

SLIGHT rises of temperature after an operation, even in cases which give no septic results, warn the surgeon that he is probably erring in some way, and that his methods and material should be pretty thoroughly overhauled.—*International Journal of Surgery.*

